# Ethical considerations in the biomedical research: analysis of national biomedical research ethics guidelines in Iran

**Published:** 2019-04-17

**Authors:** Amirhossein Mardani, Maryam Nakhoda, Alireza Noruzi, Ehsan Shamsi Gooshki

**Affiliations:** 1 *PhD Student in Information Science & Knowledge Study, * *Department of Information Science & Knowledge Studies, * * Faculty of Management, University of Tehran, Tehran, Iran.*; 2 *Assistant Professor, Department of Information Science & Knowledge Studies, Faculty of Management, University of Tehran, Tehran, Iran.*; 3 *Associate Professor, Department of Information Science & Knowledge Studies, * *Faculty* * of Management, University of Tehran, Tehran, Iran.*; 4 *Assistant Professor, Medical Ethics and History of Medicine Research Center, Tehran University of Medical Sciences, Tehran, Iran; Department of Medical Ethics, School of Medicine, Tehran University of Medical Sciences, Tehran, Iran.*

**Keywords:** Research ethics, Research process, Ethical considerations, Research ethics guideline

## Abstract

The national guidelines for biomedical research ethics are approved by the “National Committee for Ethics in Biomedical Research” at the Iranian Ministry of Health and Medical Education as the regulatory body for biomedical research in the country. The focus of these guidelines should be on the ethical issues related to different stages of the research process, which would lead to increased research integrity and better supervision of research activities. The present study analyzed the contents of these national guidelines to clarify the ethical considerations connected to the five stages of a research process including 1) proposing, 2) approval, 3) operation, 4) documentation and 5) publishing. The findings showed that the assessed guidelines laid more emphasis on the ethical considerations related to the research operation stage rather than the proposal stage. In other words, activities such as identification of the research problem, formulation of hypotheses and questions, financial evaluation, data analysis and data interpretation did not receive adequate attention in these guidelines. Most of the guidelines presented subject categories such as the rights of participants and supervisory considerations in the “research operation stage”, ethical considerations in the “evaluation and approval procedure stage”, and editorial responsibilities in the “research review and publication stage”. In general, despite noticeable content for guiding researchers for ethical conduction of research the national guidelines are not adequately developed to cover comprehensive and sufficient ethical considerations regarding all the activities of research.

## Introduction

Ethical codes stipulated in research ethics standards include responsibilities and considerations that are set to prevent violation of research integrity during the research process. These ethical considerations must be extended to all research activities from research design to publication (1), because every stage of the research process is prone to irresponsible behaviors and research misconducts, which can cause deviations in research activities (2, 3). Despite the development of standards for research ethics, however, researchers encounter additional challenges that are not specifically addressed in the standards; such inadequacies may jeopardize research integrity and increase the risk of misconducts in all stages of the research process (4).

Thus, one of the most important functions of research systems is to develop ethical standards for all research activities from research design to publication (5). Due to the significance of following these standards throughout research stages, most developed countries exercise systematic supervision over all stages of the research process (6). The Iranian Ministry of Health and Medical Education (MOHME) has approved national guidelines for biomedical research ethics as a comprehensive framework of ethical considerations to improve research quality. These guidelines focus on the necessity and importance of observing research ethics standards not only in publication, but in all research stages (7).

**Figure 1:**
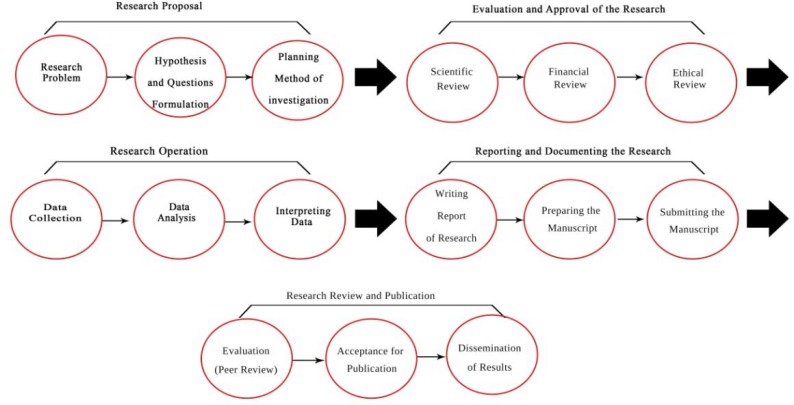
Stages and steps of biomedical research process (8)

The ultimate goal of research ethics standards, including guidelines and instructions, is to develop and modify a comprehensive framework for ethical responsibilities and considerations in research (7). Therefore, assessing the content of officially approved guidelines for research ethics allows us to understand the present state of the considerations already issued by MOHME as the main authority. A number of studies have been conducted to investigate the research ethics in Iran and have proposed ethical considerations for different stages of the research process (1, 5), but they have not addressed the ethical considerations related to activities in biomedical research. The present study, however, tried to investigate the research ethics guidelines approved by MOHME to assess implementation of the ethical considerations in various stages of the research process. In other words, it aimed to determine which activities of the research process have received more attention in the guidelines, and what ethical considerations pertain to each stage of research. In order to perform a solid and accurate analysis of the ethical considerations related to research stages in the guidelines for research ethics, it is essential that we acquire a deep understanding of the research process in the biomedical research environment in Iran. Previously (8), we described the process of biomedical research in five stages and fifteen steps (Figure 1), and the present study adopted this framework to investigate ethical considerations in research.

## Methods

For a textual investigation of research ethics standards, a qualitative content analysis was used to examine the text of the codes and general and specialized guidelines approved by MOHME. Additionally, the concept of a multi-stage research process was used to formulate the ethical considerations in research (5). Stages of the research process in Figure 1 were considered as predefined categories for recording information. Because of these predefined categories, we employed directed content analysis, which is more systematic than other methods of content analysis (9). The units of analysis included concepts of the ethical considerations stipulated in the ethical codes of the guidelines, where information such as ethical considerations is categorized based on the stages of the research process. Ethical considerations included behaviors that one must or must not engage in and are clearly pointed out in the codes of ethical conducts (10), for instance, the obligation of researchers to fully inform research participants about the benefits and risks of their study. Accordingly, we tried to extract the concepts and considerations implicitly mentioned in the ethical codes and match these concepts with the stages of the research process.

Open and axial coding were used to extract and collect data. In open coding, the information stipulated in the ethical codes was segmented into independent concepts. The results of open coding are shown in Table 2. The extracted concepts of the ethical considerations are categorized in the steps of the research process as depicted in Table 2. For axial coding, the results of open coding were reorganized and subdivided into subcategories. The results of axial coding are shown in Table 3, where the stages of the research process are presented as the main categories, and issues such as financial contracts or the rights of research participants as the subcategories. Finally, two medical ethics experts who were familiar with research ethics guidelines and the coding process were asked to evaluate the validity of the data collection and analysis.

## Results

In this study, thirteen documents, all in Farsi and available through the website of the “National Committee for Ethics in Biomedical Research of Iran” were reviewed for content analysis during 2017 (Table 1). 

**Table 1:** Approved biomedical research ethics guidelines and instructions by MOHME

Number	Guidelines	Year	Available Links
**1**	National publications ethics guidelines	2009	http://ethics.research.ac.ir/docs/pu blication_guideline.pdf?cbt=2
**2**	General ethics guidelines for medical research including human subjects	2013	http://ethics.research.ac.ir/docs/ge neral.doc
**3**	Instructions for performing multi- central research	2014	http://ethics.research.ac.ir/docs/m ulti_central.pdf
**4**	Instructions for using health information and data	2014	http://ethics.research.ac.ir/docs/dat a_access9.doc
**5**	Guidelines for approval process of clinical trial protocols for new drug registration by the Iranian Food and Drug Organization (FDO)	2014	http://ethics.research.ac.ir/docs/bi oequivalence6.pdf
**6**	Guidelines for administration of clinical trials in association with pharmaceutical and medical equipment companies and other private firms	2014	http://ethics.research.ac.ir/docs/pi- responsibility-7.pdf
**7**	Instructions on investigation of research misconduct	2009	http://ethics.research.ac.ir/docs/res earch_misconduct_guideline.pdf
**8**	Instructions for formulation, categorization and description of responsibilities of ethics committees in biomedical research	2013	http://ethics.research.ac.ir/docs/eth ics.pdf
**9**	The specific ethics guidelines for stem cell research	2016	http://ethics.research.ac.ir/docs/ste m_cell.doc
**10**	The specific ethics guidelines for research on human organs and tissues	2015	http://ethics.research.ac.ir/docs/tis sue_and_organ.doc
**11**	The specific ethics guidelines for research on vulnerable groups	2013	http://ethics.research.ac.ir/docs/vu lnerables.doc
**12**	The specific ethics guidelines for performing clinical trials	2013	http://ethics.research.ac.ir/docs/cli nical_trial.doc
**13**	National instructions for patient- funded clinical trials	2013	http://ethics.research.ac.ir/docs/pat ient_funded_clinical_trials-11.pdf

Overall, 161 ethical considerations were identified and categorized based on their concepts and subjects in the five stages of the research process. Clearly, there is a larger number of ethical codes in the text of the research ethics guidelines. However, the ethical considerations were identified and categorized according to the subjects mentioned in one or more ethical codes. Ultimately, duplicate and similar codes were merged.

***1. Research Proposal***

Analysis of the standards indicated that the concepts related to the step ‘identification of the research problem’ had only been mentioned in two ethical codes: 1) “Others’ ideas may not be used without observing the intellectual property rights” in the instructions on investigation of research misconduct; and 2) “The main purpose of every research is to promote human health while respecting human rights and dignity” in the general ethics guidelines for medical research including human subjects. The ‘hypotheses and questions formulation’ step had not been addressed in the standards. Furthermore, researchers need to examine ethical considerations related to the research method and design in the step of ‘planning the method of investigation’; however, the ethical codes related to this step generally focused on the rights of participants (informed consent, confidentiality of personal information, and follow-up), facilitation of research supervision (location and setting) and registering the proposals. Moreover, researchers need to take into account certain financial issues at the time of planning the study. Aside from financial priorities that should be considered in research centers and university committees, the ethical codes only involved refraining from accepting unfair conditions by financial sponsors regarding publication of results, and declaring conflict of interests in financial contracts (Table 2).

**Table 2:** Ethical considerations related to “Research Proposal”

Research Step	Related Considerations Stipulated in the Ethical Codes	Guideline(s)
Research Problem	Observing intellectual property rights when using research ideas proposed by others in proposals	1,2,3,7,9,12
	Promoting human health while respecting human dignity and rights as the main aim of research	1,2,3,7,9,12
Planning Research Design	Observing publication ethics standards, ethical codes and research regulations in preparing research proposals	1,2,3,7,9,12
	Conducting research based on a pre-defined proposal	1,2,3,7,9,12
	Preparing protocols for clinical trials	1,2,3,7,9,12
	Preparing informed consent forms	1,2,3,7,9,12
	Predicting and introducing a clearly defined protocol to ensure confidentiality and security of information	1,2,3,7,9,12
	Determining and predicting follow-up with human subjects after study completion	1,2,3,7,9,12
	Determining the exact location of the research site in the research proposal	1,2,3,7,9,12
	Registering the proposals for clinical trials on the IRCT	1,2,3,7,9,12
	Following official criteria and ethical codes while signing a contract with financial sponsors	1,2,3,7,9,12
	Declining the financial sponsor's condition to remove or not publish undesirable findings in the contract between the researcher(s) and the financial sponsor	1,2,3,7,9,12
	Declining the financial sponsor’s condition to not declare conflict of interests in the research report in the contract between the researcher(s) and the financial sponsor	1,2,3,7,9,12

***2. Evaluation and Approval of the Research***

Unlike the previous stage, the main actors in this stage are the committees responsible for scientific evaluation of proposals based on peer review process, financial evaluation, and finally ethical evaluation. Research ethics committees conduct an evaluation of research proposals prior to issuing an ethics approval. Therefore, the confidentiality of personal information is taken into account when dealing with research misconduct, conflict of interests among committee members, privacy and confidentiality of participants, and cooperation of other researchers for accountability. As a general rule, issues related to ‘research financial evaluation’ are addressed by the committee for scientific evaluation to determine priorities in attracting financial support. The studied documents did not consider such matters. Although these financial issues and criteria were included in the assessed standards, concepts such as disclosure of financial resources by researchers and attention to criteria of financial contracts as well as requirements of financial sponsors were mentioned in ‘ethics evaluation’. In addition, the codes related to ‘scientific evaluation’ were very rare and limited to research methodology and conflict of interests among members of scientific associations. (Table 3). 

**Table 3:** Ethical considerations related to “Evaluation and Approval of the Research”

Research Step	Related Considerations Stipulated in the Ethical Codes	Guideline(s)
Scientific Evaluation	Evaluating and approving the scientific aspects and methodology of research proposals	1,2,3,5,6,7,8,9,11,12,13
	Disclosing conflict of interests of members of the scientific committee in relationship to the researchers and financial sponsors	1,2,3,5,6,7,8,9,11,12,13
Ethical Evaluation	Submitting proposals to the ethics committee to achieve a certificate	1,2,3,5,6,7,8,9,11,12,13
	Evaluation and approval of the research proposal by the research ethics committee	1,2,3,5,6,7,8,9,11,12,13
	Evaluation and approval of clinical protocols by the research ethics committee	1,2,3,5,6,7,8,9,11,12,13
	Appointment of a qualified supervisor by the research ethics committee	1,2,3,5,6,7,8,9,11,12,13
	Ensuring the research ethics committee will enforce and implement information protection and confidentiality	1,2,3,5,6,7,8,9,11,12,13
	Evaluation of the potential profit-loss ratio for human subjects by the research ethics committee	1,2,3,5,6,7,8,9,11,12,13
	Observance of confidentiality of complaint proceedings through the end by the research ethics committee when dealing with research misconduct and deviations	1,2,3,5,6,7,8,9,11,12,13
	Providing research data and evidence to the research ethics committee by researchers	1,2,3,5,6,7,8,9,11,12,13
	Informing the research ethics committee of changes in protocol and proposal by researchers	1,2,3,5,6,7,8,9,11,12,13
	Declaring conflict of interests between members of the research ethics committee, and researchers and financial sponsors	1,2,3,5,6,7,8,9,11,12,13
	Obligation of the research ethics committee to address limitations in publishing research results in the contract between researchers and financial sponsors	1,2,3,5,6,7,8,9,11,12,13
	Establishing the scientific and technical competencies of researchers to design and conduct research	1,2,3,5,6,7,8,9,11,12,13
	Adaptation of research methods to social, cultural and religious values of human subjects and the society	1,2,3,5,6,7,8,9,11,12,13
	Disclosure of financial sponsors’ identity by researcher	1,2,3,5,6,7,8,9,11,12,13
	Declaring conflict of interests among the researchers in a research team	1,2,3,5,6,7,8,9,11,12,13

***3. Research Operation***

The codes related to ‘data collection’ focused on accessibility and confidentiality of the information collected from participants, informed consent, protection of study subjects, and supervision of the study. Text of the standards for other stages including ‘data analyses and ‘data interpretation’ did not contain any specific details (Table 4).

**Table 4:** Ethical considerations related to “Research Operation”

Research Step	Related Considerations Stipulated in the Ethical Codes	Guideline(s)
Data Collection	Clarification and facilitation of access to health data	1,2,3,4,6,7,8,9,10,11,12
	Not requesting a share of research (such as author’s rights) solely due to ownership and provision of health data	1,2,3,4,6,7,8,9,10,11,12
	Sharing health data	1,2,3,4,6,7,8,9,10,11,12
	Appropriate registration, application and storage of health data	1,2,3,4,6,7,8,9,10,11,12
	Maintaining privacy in providing health information to researchers by health institutions or health databases	1,2,3,4,6,7,8,9,10,11,12
	Respecting the privacy and preserving the confidentiality of human subjects’ information	1,2,3,4,6,7,8,9,10,11,12
	Providing human subjects with any information that may affect their decision to take part in research	1,2,3,4,6,7,8,9,10,11,12
	Informing human subjects of changes in the research process as well as new findings related to the advantages and risks of interventions in research	1,2,3,4,6,7,8,9,10,11,12
	Obtaining in-time and clear informed consent from human subjects	1,2,3,4,6,7,8,9,10,11,12
	Obtaining informed consent from human subjects for publishing their data	1,2,3,4,6,7,8,9,10,11,12
	Providing human subjects with the freedom to accept or refuse participation in the research	1,2,3,4,6,7,8,9,10,11,12
	Providing human subjects with the necessary motivation or reward to take part in the research	1,2,3,4,6,7,8,9,10,11,12
	Ensuring the health and security of human subjects during and after research	1,2,3,4,6,7,8,9,10,11,12
	Preserving the human dignity, respect, and physical and mental integrity of research participants	1,2,3,4,6,7,8,9,10,11,12
	Paying attention to the personal, cultural and religious differences of research participants	1,2,3,4,6,7,8,9,10,11,12
	Avoiding prejudice toward a special group of people	1,2,3,4,6,7,8,9,10,11,12
	Taking care of vulnerable groups	1,2,3,4,6,7,8,9,10,11,12
	Conducting research in a place with precise control facilities	1,2,3,4,6,7,8,9,10,11,12
	Informing the research ethics committee of changes in research proposal and protocol	1,2,3,4,6,7,8,9,10,11,12
	Supervision and monitoring of the research by the research ethics committee	1,2,3,4,6,7,8,9,10,11,12
	Providing the research ethics committee with periodical reports of the research progress	1,2,3,4,6,7,8,9,10,11,12
	Adherence of the researcher to contents of the research proposal	1,2,3,4,6,7,8,9,10,11,12

***4. Reporting and Documenting the Research***

This stage involves issues such as referencing, copyrights, data falsification and fabrication, and precise and real-time reporting of the results to all beneficiaries in the form of ethical codes related to the ‘reporting style’. It also comprises consideration of authors’ rights and criteria, authors’ accountability for accuracy and ethics conformity of research content, disclosure of organizational dependencies, and conflict of interests among authors for the step of ‘preparing a copy of the research’. Codes related to ‘submission to journals’ deal with deviations such as publication of overlapping materials and duplicate publication (Table 5).

**Table 5:** Ethical considerations related to “Reporting and Documenting the Research”

Research Step	Related Considerations Stipulated in the Ethical Codes	Guideline(s)
Writing the Research Report	Avoiding reference to oneself or others for the sole purpose of increasing the number of irrelevant references	1,2,7,8,9,10,12
	Asking the permission of intellectual property owners to use tables, questionnaires and texts	1,2,7,8,9,10,12
	Mentioning the reference when citing a text or its translated counterpart, conclusion, summary or ideas	1,2,7,8,9,10,12
	Making citations in agreement with the intentions of the original authors	1,2,7,8,9,10,12
	Avoiding verbatim or partial plagiarism from articles or proposals by other authors	1,2,7,8,9,10,12
	Avoiding data fabrication and falsification in research	1,2,7,8,9,10,12
	Preserving the confidentiality and privacy of individuals and their information when publishing research results	1,2,7,8,9,10,12
	Publishing and making research results available in an accurate, timely and appropriate manner for other stakeholders	1,2,7,8,9,10,12
	Disclosure of negative and positive results as well as the side effects of the research	1,2,7,8,9,10,12
	Avoiding authors from limiting the publication of research results due to issues such as sensitivity of the study participants, negative social consequences, or financial sponsors requests	1,2,7,8,9,10,12
Preparing the Manuscript	Avoiding author’s name fabrication (i.e., removing qualified names and adding unqualified names)	1,2,7,8,9,10,12
	Observing the author’s rights based on the four authoring conditions	1,2,7,8,9,10,12
	Observing the order of the authors’ names based on their level of participation and by collective agreement	1,2,7,8,9,10,12
	Informing all contributors and adjusting the final manuscript to include their names in the authors’ list	1,2,7,8,9,10,12
	Selecting the contributor with the largest share in the presentation of the research idea as the first author	1,2,7,8,9,10,12
	Accountability of all authors regarding accuracy of the manuscript	1,2,7,8,9,10,12
	Assuming responsibility in terms of the general and specialized research ethics guidelines in research reports	1,2,7,8,9,10,12
	Authors’ assurance of the capability of all persons involved in the research	1,2,7,8,9,10,12
	Avoiding services such as those that type hand-written texts and submit manuscripts to journals	1,2,7,8,9,10,12
	Disclosing the organizational affiliation of authors	1,2,7,8,9,10,12
	Mentioning authors’ affiliation when submitting the manuscript to a journal	1,2,7,8,9,10,12
	Disclosing the financial resources of the research	1,2,7,8,9,10,12
	Declaring any potential conflict of interests among manuscript authors	1,2,7,8,9,10,12
	Declaring any conflict of interests arising from the contract between the researchers and financial sponsors	1,2,7,8,9,10,12
Submitting the Manuscript	Considering the authors’ freedom to select scientific journals for the publication of their articles	1,2,7,8,9,10,12
	Informing the journal to cancel publication before approval of the manuscript	1,2,7,8,9,10,12
	Avoiding duplicate submission and publication of overlapping manuscripts in different journals	1,2,7,8,9,10,12
	Informing editors of journals about reprint of a paper published in another languages	1,2,7,8,9,10,12

***5. Research Review and Publication***

Activities related to this stage are concerned with responsibilities of journals. Technical and scientific competencies, confidentiality and privacy of the reviewing process, conflict of interests among reviewers and editors, as well as investigation and reporting of research misconduct are all the focus of ‘peer review’. The ‘acceptance for publication’ step deals with issues such as independency of the journal, confidentiality of the identities of study participants, and the possibility of publishing undesirable results. The codes related to the ‘publication of research’ step revolve around issues such as the possibility of criticizing recently published articles and publishing undesirable results (Table 6).

**Table 6:** Ethical considerations related to “Research Review and Publication”^1^

Research Step	Related Considerations Stipulated in the Ethical Codes
Peer Review	Selecting individuals with the necessary scientific and technical ability to review manuscripts
	Informing the journal editor of a lack of the scientific and technical qualifications necessary for reviewing manuscripts
	Providing reviewers with the required information and guidelines
	Declaring any conflict of interests in the reviewing of manuscripts
	Introducing the sponsors of the journal
	Declaring conflict of interests in the reviewing of manuscripts by the editorial board members
	Preserving the confidentiality of the manuscript by the reviewer
	Respecting privacy and confidentiality at all stages of the manuscript review by the editor
	Avoiding usage of reviewed manuscripts for other purposes
	Asking the editor’s permission to consult others about reviewed manuscripts
	Preventing potential communication between manuscript authors and reviewers
	Respect for the intellectual independence of manuscript authors by the reviewer
	Reviewing the manuscripts on the preset date announced by the editor
	Fair and impartial review and prioritization of the manuscripts considering only scientific and technical qualities
	Ensuring the receipt of the research ethics code and registration of the clinical trial related to the article
	Notifying the editor of any failure to observe the provisions of the guidelines and ethical codes in the manuscript
	Investigating possible occurrences of research misconduct and deception in the received manuscripts and meting out appropriate treatment
	Reporting occurrence of research misconduct while maintaining maximum confidentiality to the research ethics committee, or the director/head of the authors’ place of work, or the education department if the authors fail to provide a convincing explanation
	Addressing complaints about the submission process or other stages in the procedures required by the journal
Acceptance for Publication	Respecting the editor’s independence in accepting or rejecting manuscripts
	Ensuring confidentiality of the information of individuals and patients following publication of the article
	Referring issues and decision-making about publishing the identity of participants in the study to the research ethics committee
	Not forcing the authors to cite studies published in the previous volumes of the journal
	Asking the corresponding author to determine the contribution of all authors involved in a study
	Providing the conditions for publication of studies that use the right methodologies and produce undesirable results
Publication of Research	Editor and editorial board member’ participation as author only in 20% of the articles of the journal
	Providing readers with the opportunity to criticize published papers
	Preventing placement of advertisements aimed at attracting readers by providing non-scientific and false information

1. *All ethical codes are related to Guideline No. 1*

In addition to classifying the considerations according to stages of the research process, an attempt was made to categorize these considerations deductively to obtain a better description of guidelines and instructions for each stage. As can be seen in Table 7 to 11, 14 categories were established for ethical considerations of each stage (Table 7-11). 

**Table 7:** Categories of the ethical considerations related to “Research Proposal”

Category	Related considerations stipulated in the ethical codes
Research Design Considerations	Observing intellectual property rights when using research ideas proposed by others in proposals
	Observing publication ethics standards, ethical codes, and research regulations in preparing research proposals
	Promoting human health while respecting human dignity and rights as the main aim of research
	Conducting research based on a pre-defined proposal
	Preparing protocols for clinical trials
	Preparing informed consent forms
	Predicting and introducing a clearly defined protocol to ensure confidentiality and security of information
	Determining and predicting follow-up on human subjects after study completion
	Determining the exact location of the research site in the research proposal
	Registering the proposals for clinical trials on the IRCT
Research Financial Contracts	Following official criteria and ethical codes while signing a contract with financial sponsors
	Declining the financial sponsor's condition to remove or not publish undesirable findings in the contract between the researcher(s) and the financial sponsor
	Declining the financial sponsor’s condition to not declare conflict of interests in the research report in the contract between the researcher(s) and the financial sponsor

**Table 8:** Categories of the ethical considerations related to “Evaluation and Approval of the Research”

Category	Related considerations stipulated in the ethical codes
Ethical Considerations	Submitting proposals to the ethics committee to achieve a certificate
	Evaluation and approval of the research proposal by the research ethics committee
	Evaluation and approval of clinical protocols by the research ethics committee
	Appointment of a qualified supervisor by the research ethics committee
	Evaluation of the potential profit-loss ratio for human subjects by the research ethics committee
	Evaluation of respecting the privacy and preserving the confidentiality of human subjects' information by the research ethics committee
	Obligation of the research ethics committee to address limitations in publishing research results in the contract between researchers and financial sponsors
	Establishing the scientific and technical competencies of researchers to design and conduct research
	Adaptation of research methods to social, cultural and religious values of human subjects and the society
Scientific Criteria	Evaluating and approving the scientific aspects and methodology of research proposals
	Disclosing conflict of interests of members of the scientific committee in relationship to the researchers and financial sponsors
Conflict of Interests for Research Evaluation	Declaring conflict of interests between members of the research ethics committee, and researchers and financial sponsors
	Disclosure of financial sponsors’ identity by researchers
	Declaring conflict of interests among the researchers in a research team

**Table 9:** Categories of the ethical considerations related to “Research Operation”

Category	Related considerations stipulated in the ethical codes
Accessibility of Health Information	Clarification and facilitation of access to health data
	Appropriate registration, application and storage of health data
	Maintaining privacy in providing health information to researchers by health institutions or health databases
	Not requesting a share of research (such as author’s rights) solely due to ownership and provision of health data
	Sharing health data
Rights of Research Participants	Providing human subjects of any information that may affect their decision to take part in research
	Informing human subjects of changes in the research process as well as new findings related to the advantages and risks of interventions in research
	Obtaining in-time and clear informed consent from human subjects
	Ensuring the health and security of human subjects during and after research
	Preserving the human dignity, respect, and physical and mental integrity of research participants
	Respecting the privacy and preserving the confidentiality of subjects’ information
	Providing the subjects with the freedom to accept or refuse participation in the research
	Providing the subjects with the necessary motivation or reward to take part in the research
	Paying attention to the personal, cultural and religious differences of research participants
	Avoiding prejudice toward a special group of people
	Taking care of vulnerable groups
Supervisory Considerations	Supervision and monitoring of the research by the research ethics committee
	Informing the research ethics committee of changes in research proposal and protocol
	Ensuring the research ethics committee will enforce and implement information protection and confidentiality
	Providing the research ethics committee with periodical reports of the research progress
	Conducting research in a place with precise control facilities
	Adherence of the researcher to contents of the research proposal
	Observance of confidentiality of complaint proceedings through the end by the research ethics committee when dealing with research misconduct and deviations
	Providing research data and evidence to the research ethics committee by researchers
	Informing the research ethics committee of changes in research protocol by researchers

**Table 10:** Categories of the ethical considerations related to “Reporting and Documenting the Research”

Category	Related considerations stipulated in the ethical codes
Author’s Task	Avoiding author’s name fabrication (i.e., removing qualified names and adding unqualified names)
	Accountability of all authors regarding accuracy of the manuscript
	Assuming responsibility in terms of the general and specialized research ethics guidelines in research reports
	Authors’ assurance of the capability of all persons involved in the research
	Informing all contributors and adjusting the final manuscript to include their names in the authors’ list
	Avoiding services such as those that type hand-written texts and submit manuscripts to journals
	Avoiding reference to oneself or others for the sole purpose of increasing the number of irrelevant references
	Informing the journal to cancel publication before approval of the manuscript
	Considering the authors’ freedom to select scientific journals for the publication of their articles
Intellectual Property Rights of Stakeholders	Observing the author’s rights based on the four authoring conditions
	Observing the order of the authors' names based on their level of participation and by collective agreement
	Selecting the individual with the largest share in the presentation of the research idea as the first author
	Mentioning authors’ affiliation when submitting the manuscript to a journal
	Mentioning the reference when citing a text or its translated counterpart, conclusion, summary or ideas
	Asking the permission of intellectual property owners to use tables, questionnaires and texts
	Making citations in agreement with the intention of the original authors
	Avoiding verbatim or partial plagiarism from articles or proposals by other authors
	Avoiding duplicate submission and publication of overlapping manuscripts in different journals
	Informing editors of journals about reprint of a paper published in another languages
	Obtaining informed consent from human subjects for publishing their data
	Preserving the confidentiality and privacy of individuals and their information when publishing research results
Clarity of Research Results	Publishing and making research results available in an accurate, timely and appropriate manner for other stakeholders
	Disclosure of negative and positive results as well as the side effects of the research
	Avoiding authors from limiting publication of research results due to issues such as sensitivity of the study participants, negative social consequences, or financial sponsors requests
	Avoiding data fabrication and falsification in research
Conflict of Interests for Authors	Declaring any potential conflict of interests among manuscript authors
	Declaring any conflict of interests arising from the contract between the researchers and financial sponsors
	Disclosing the organizational affiliation of authors
	Disclosing the financial resources of the research

**Table 11:** Categories of the ethical considerations related to “Research Review and Publication”

Category	Related considerations stipulated in the ethical codes
Editorial Responsibilities	Respecting privacy and confidentiality at all stages of the manuscript review by the editor
	Ensuring confidentiality of the information of individuals and patients following publication of the article
	Ensuring the receipt of the research ethics code and registration of the clinical trial related to the article
	Respecting editor independency in accepting or rejecting manuscripts
	Declaring conflict of interests in the reviewing of manuscripts by the editorial board members
	Introducing the sponsors of the journal
	Selecting individuals with the necessary scientific and technical ability to review manuscripts
	Providing reviewers with the required information and guidelines
	Preventing potential communication between manuscript authors and reviewers
	Fair and impartial review and prioritization of the manuscripts considering only scientific and technical qualities
	Asking the corresponding author to determine the contribution of all the authors involved in a study
	Providing the conditions for publication of studies that use the right methodologies and Produce undesirable results
	Not forcing the authors to cite studies published in the previous volumes of the journal
	Providing readers with the opportunity to criticize published papers
	Addressing complaints about the submission process or other stages in the procedures required by the journal
	Editor and editorial board member’ participation as author only in 20% of the articles of journal
	Preventing placement of advertisements aimed at attracting readers by providing non-scientific and false information
	Investigating possible occurrences of research misconduct and deception in the received manuscripts and meting out appropriate treatment
	Reporting the occurrence of research misconduct while maintaining maximum confidentiality to the research ethics committee, or the director/head of the authors’ place of work, or the education department if the authors fail to provide a convincing explanation
	Referring issues and decision-making about publishing the identity of participants in the articles to the research ethics committee
Reviewer Responsibilities	Having the scientific and technical qualifications necessary for reviewing manuscripts
	Informing the journal editor of a lack of the scientific and technical qualifications necessary for reviewing manuscripts
	Declaring any conflict of interests in the reviewing of manuscripts
	Preserving the confidentiality of the manuscript by the reviewer
	Avoiding usage of reviewed manuscripts for other purposes
	Asking the editor’s permission to consult others about reviewed manuscripts
	Respect for the intellectual independence of manuscript authors by the reviewer
	Reviewing the manuscripts on the preset date announced by the editor
	Notifying the editor of any failure to observe the provisions of the guidelines and ethical codes in the manuscript

**Figure 2:**
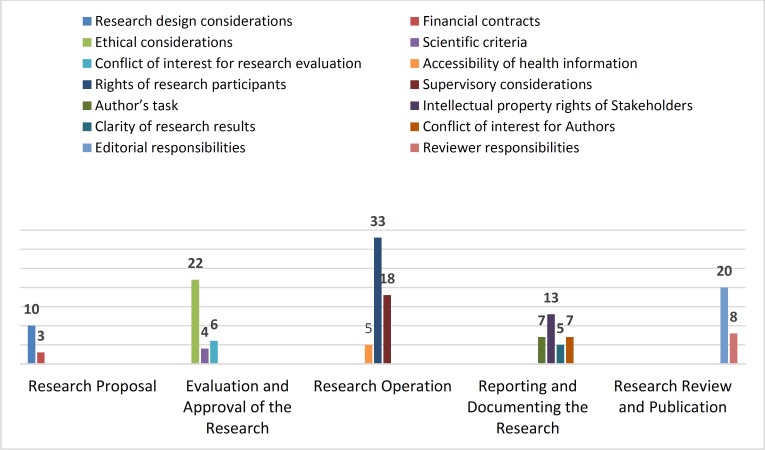
Distribution of the ethical considerations categories in the research process

The following categories were, respectively, significantly prominent in the guidelines and instructions: 1) ‘rights of research participants’ (33 items from 161 ethical considerations: 20.49%) related to the stage of ‘research operation’; 2) ‘ethical considerations’ (22 items: 13.66%) related to the stage of ‘evaluation and approval of the research; 3) ‘editorial responsibilities’ (20 items: 12.42%) related to the stage of ‘research review and publication’; and 4) ‘supervisory considerations’ (18 items: 11.18%) related to the stage of ‘research operation’. Moreover, issues related to financial contracts, scientific criteria for evaluation and approval of research, accessibility of health information for researchers and clarity of research results were emphasized in guidelines and instructions (Figure 2).

With 56 considerations related to the ‘research operation’ stage, it is the most highlighted stage in terms of attention to ethical considerations in the guidelines and instructions for various stages of the research process. On the contrary, ‘research proposal’ is the least prominent stage with just 13 considerations pertaining to it. Moreover, in the stage of ‘reporting and documenting the research’, editors of research ethics guidelines and instructions focus on different issues in different categories.

## Discussion

The guidelines for biomedical research ethics offered by MOHME as the main authority may serve as a means to prevent research deviations and enhance supervision over research activities. However, our findings showed that the existing ethical considerations are not adequately developed to cover different activities in various stages of the research process. These guidelines do not provide comprehensive and sufficient insight about the ethical responsibilities and considerations related to all activities of the research process.

Research ethics guidelines have mainly focused on the stages of research operation, reporting and publication. One reason for this could be the dominant approach of corresponding authorities to the approval and supervision of the research activities related to these stages. Furthermore, prevention and control of research misconducts are not supervised continuously during the research process (11). As the stage of ‘research review and publication’ is more vulnerable to misconduct, it has received a lot of attention in the form of supervisory measures. Evident manifestations of research misconduct have received the most supervision by research ethics committees in universities and have been especially emphasized in research ethics standards. They are mainly comprised of deviations in the reporting and publication stages including plagiarism, data fabrication, data falsification, re-publication and overlapping of research results. Nevertheless, research misconduct constitutes an expansive range of irresponsible behaviors and is not just limited to the reporting and publication stages (12, 13). Thus, it seems that in dealing with various representations of misconduct in the research process, it would be better to benefit from a more practical solution in view of the ethical considerations in research ethics guidelines.

The study results showed that the majority of these guidelines pay great attention to the rights of research participants including informed consent, ensuring the health and security of human subjects, preserving human dignity, subjects’ physical and mental integrity, maintaining confidentiality of the subjects’ information, and taking care of vulnerable groups. Research standards emphasize free and informed consent as well as a net balance of benefits over harms for all participants (14). Consistent with present findings, a study on the guidelines, regulations, and ethical codes of Arab countries in the Middle East by Alahmad et al. indicated that scientific validity and obligations for informed consent, confidentiality, and the balance of benefits and risks are mentioned in most of the documents (15).

In the examined research ethics standards, ethical considerations about pre-research activities such as research motives, research problems, and planning research design were less emphasized. There were very limited ethical codes related to research problems, which could be due to intangibility of some activities, and the low potential of certain issues for supervision and monitoring at this stage. Thus, research should be conducted based on the needs of health-care providers, and this is an ethical obligation for researchers also highlighted in research ethics guidelines and instructions. Notwithstanding, this matter has been neglected in research ethical codes. In other words, the relationship between research, the researcher and community research needs are overlooked. Lack of research in the field of health and medical sciences based on national and local needs and insufficient application of national research results are among the most notable problems that affect the effectiveness and efficiency of research in this domain (16). Therefore, it seems that ethical codes could serve as an indirect and useful intervention in the process of conducting research based on local needs. Indeed, in addition to a lack of need-based research, the proclivity of faculty promotion regulations and other research protocols toward quantitative measures may solely motivate researchers toward research results publication and earning quantitative privileges (17, 18). 

Another important issue that requires more attention is related to financial contracts. Some cases of research misconduct have been reported following the demand and pressure of financial sponsors for publishing desirable results. This is because the conditions and shares of parties (researchers and financial sponsors) are not clarified in contracts, and research ethics committees do not examine the contracts meticulously enough (19, 20). It is necessary to define and clarify the conditions and shares of financial sponsors in research ethical codes. Another important point in research guidelines is the accessibility of data and information for researchers. Limitations in existing research ethics guidelines have subjected researchers to situations where owners of data or equipment make unjust and unconventional demands in return for access to information. Therefore, it is essential that officials and administrators develop independent guidelines and instructions to clarify the conditions and requirements for sharing health data and accessibility of data for research stakeholders. 

Lack of ethical codes in the ‘review of research proposal’ stage is another issue that should be taken into account. The challenging issue that still remains unresolved is whether or not research ethics committees should attend the sessions for scientific evaluation of proposals. The scientific criteria mentioned in ethical codes are limited to methodology of research proposals and conflict of interests among research committees. It is necessary that the scientific review be conducted strictly because much of the deviations in the following stages including inappropriate criteria for inclusion and exclusion of participants or lower statistical power occur because of incorrect projection of the research process. The necessity for involvement of methodology and statistics experts in research ethics committees also attests the importance of scientific standards in research evaluation and approval. Generally speaking, scientific and ethical evaluations cannot be separated, and the research ethics committee naturally needs to consider these two simultaneously. According to the Declaration of Helsinky, medical research on human beings should follow approved and scientific principles and be based on comprehensive knowledge of scientific texts, related resources, laboratory experimentations and animal trials if necessary (21). Furthermore, failure to address issues of research methodology is evident in some activities in the ‘research operation’ stage. There was no ethical code for data analysis and data interpretation in the guidelines. In the end, it should be added that activities including quality control of research data or careful handling of statistical premises that entail serious ethical considerations may lead to research misconduct (22).

## Conclusion

The contents of research ethics guidelines affect their interpretation and application, and therefore modifying and improving them can enhance their usage, prevent research misconducts and improve supervision of the research process. Although national research ethics guidelines stress the necessity of research integrity, a current evaluation of the guidelines approved by MOHME indicated that ethical considerations are not adequately developed to cover different activities involved in various stages of the research process. In order to implement a systematic supervision approach throughout the research process, it is necessary that responsible authorities revise and modify the existing research ethics guidelines. Thus, research ethics committees will consider these responsibilities and considerations in their supervisory function, and other stakeholders will be informed about the ethical considerations related to them. 

## Notes:

**Citation to this article:**

Mardani AH, Nakhoda M, Noruzi A, Shamsi Gooshki E. Ethical considerations in the biomedical research: analysis of national biomedical research ethics guidelines in Iran. J Med Ethics Hist. 2019; 12: 4.
